# Diclofenac Versus Ketorolac for Pain Control After Primary Total Joint Arthroplasty: A Comparative Analysis

**DOI:** 10.7759/cureus.7310

**Published:** 2020-03-18

**Authors:** Nicole E George, Cheryle Gurk-Turner, Nequesha S Mohamed, Wayne A Wilkie, Ethan A Remily, Iciar M Dávila Castrodad, Elana Roadcloud, Ronald Delanois

**Affiliations:** 1 Orthopedic Surgery, Aultman Hospital, Canton, USA; 2 Pain Management, Lifebridge Health-Rubin Institute for Advanced Orthopedics, Baltimore, USA; 3 Orthopedics, Lifebridge Health-Rubin Institute for Advanced Orthopedics, Baltimore, USA; 4 Orthopedic Surgery, Hackensack Meridian School of Medicine at Seton Hall University, Nutley, USA

**Keywords:** diclofenac, ketorolac, total hip arthroplasty, total knee arthroplasty, pain control

## Abstract

Introduction

As total hip arthroplasty (THA) and total knee arthroplasty (TKA) transition to outpatient settings, appropriate pain management remains a challenge. Nonsteroidal anti-inflammatory drugs (NSAIDs) may subvert the need for postoperative opioids. This study evaluated: 1) total opioid consumption; 2) postoperative pain intensity; 3) discharge destination; 4) length of stay (LOS); and 5) THA and TKA patients’ satisfaction in receiving adjunctive intravenous (IV) diclofenac or ketorolac.

Methods

In this retrospective cohort study, patients scheduled to undergo primary THA or TKA by a single surgeon between March 2017 and April 2018 were identified. Patients were stratified based on the receipt of IV diclofenac (THA: n = 25; TKA: n = 51) or IV ketorolac (THA: n = 28; TKA: n = 32) in addition to the standard pain management regimen. Student’s t-testing and Chi-square were used to analyze continuous and categorical variables, respectively.

Results

TKA diclofenac patients had lower opioid consumption 12 hours postoperatively (p: 0.037). TKA patients in the diclofenac cohort were discharged to home less often (p: 0.025). Both diclofenac cohorts had greater patient satisfaction than the ketorolac cohorts (p: <0.05). There was no significant difference between groups in postoperative pain intensity at 24 or 48 hours or in the length of stay (p: >0.05 for all).

Conclusion

This study demonstrated that both TKA and THA patients treated with IV diclofenac had no difference in postoperative pain intensity while THA patients had no difference in opioid consumption relative to those treated with IV ketorolac. Further comparison of IV NSAIDs with other IV pain medications may provide broader insight into the ideal management for postoperative pain for this widening patient population.

## Introduction

Total joint arthroplasty (TJA) poses a significant economic burden to the healthcare system, with combined inpatient costs for Medicare total hip and knee arthroplasties exceeding USD 7 billion in 2014 [1]. Innovations and improvements in total knee arthroplasty (TKA) and total hip arthroplasty (THA) have helped reduce the burden of postoperative care required for both and combat the rising cost of care [2,3]. As a result, TKA was removed from the list of inpatient-only procedures by the Centers for Medicare & Medicaid Services (CMS) in 2018, with THA to follow in 2020 [4]. As the demand for TKA and THA continues to increase, the anticipated volume shift to outpatient centers may assist in reducing healthcare expenditure [5]. Still, the challenges associated with optimizing outcomes such as pain and satisfaction for TJA patients remain [6]. Despite best efforts, postoperative pain continues to pose a challenge for many surgical care teams, which may impede the transition to outpatient care [7,8]. Current pain management protocols have been extensively scrutinized to identify opportunities to maximize clinical outcomes and reduce postoperative complications [9-11]. However, the inclusion of opioid medications in such protocols has been associated with adverse outcomes that can be avoided [12].

Although opioids have classically been a mainstay in the management of postsurgical pain, cognizance of their unfavorable side-effect profile and potential to prolong hospital stays has spurred initiatives to identify alternative pain-control avenues [12-14]. A number of different agents and modalities have been studied as alternative pain therapies, including local or regional nerve blocks, acetaminophen, gabapentin, and nonsteroidal anti-inflammatory drugs (NSAIDs). In particular, NSAIDs have shown promise at maximizing analgesia while decreasing the opioid-related side effects and addiction risk [15-20]. Additionally, intravenous (IV) preparations have the benefit of greater bioavailability than oral medication for patients in need of acute pain management [21,22]. As opioid prescriptions are increasingly restricted and controlled, IV NSAID medications like ketorolac and diclofenac may be well suited to acutely decrease postoperative pain, increasing patient satisfaction, and facilitating earlier discharge in TJA patients [23,24].

Although several studies have evaluated the efficacy of IV ketorolac and diclofenac on postoperative outcomes and pain control, there is a paucity of literature comparing the two in the setting of primary TJA. As such, this study evaluated: 1) total opioid consumption; 2) postoperative pain intensity; 3) discharge disposition; 4) length of hospital stay; and 5) satisfaction with adjunctive IV diclofenac or ketorolac pain control in primary THA and TKA patients, at a single institution between 2017 and 2018.

## Materials and methods

Patient selection

Institutional Review Board approval was obtained for all aspects of this retrospective cohort study. All patients that underwent TJA with a single surgeon between March 1, 2017 and April 31, 2018 were identified and screened for study inclusion. Patients were included if they had undergone a primary TKA or THA and received adjunctive diclofenac or ketorolac as a part of their postoperative pain management regimen. Patients were excluded from the review if they were on methadone therapy, had a history of chronic opioid use, had a low calculated creatinine clearance (less than 30 mL/min2), had undergone epidural anesthesia during their procedure, had a poor cardiac function, had an allergy to any NSAIDs, narcotics, or local anesthetics used in the study, or if they had undergone revision or conversion THA or TKA.

We identified a total of 136 eligible TJA patients who had received IV diclofenac or IV ketorolac that were blinded to their perioperative pain regimen. The final patient cohorts comprised 25 THA patients and 51 TKA patients who had received postoperative IV diclofenac, and 28 THA patients and 32 TKA patients who had received postoperative IV ketorolac.

Pain regimen

The standard pain regimen included: celecoxib 400 mg per oral (PO) preoperatively, then celecoxib 200 mg twice per day, starting on the evening of postoperative day one (POD1) (Table 1).

**Table 1:** Perioperative multimodal pain control regimen for the intervention groups TKA: total knee arthroplasty; THA: total hip arthroplasty; BID: twice a day; IV: intravenous; PACU: postoperative anesthesia care unit; PO: per oral; POD: postoperative day; PRN: as needed

THA	Diclofenac cohort	Ketorolac cohort
Preoperative	Celecoxib 400 mg PO (dose-adjusted for age and/or renal dysfunction) ±spinal anesthesia (with morphine and bupivacaine)	Celecoxib 400 mg PO (dose-adjusted for age and/or renal dysfunction) ±spinal anesthesia (with morphine and bupivacaine)
Intraoperative	Periarticular multimodal drug injection containing bupivacaine, epinephrine, dexamethasone, ketorolac, and morphine	Periarticular multimodal drug injection containing bupivacaine, epinephrine, dexamethasone, ketorolac, and morphine
Postoperative	Diclofenac sodium 37.5 mg IV q6 hours x4 doses (upon PACU arrival); celecoxib 200 mg PO BID (beginning evening of POD1); oxycodone 5/10 mg PO q3 hours PRN for moderate/severe pain; hydromorphone 0.2/0.4 mg IV q5 minutes PRN for moderate/severe pain (PACU only); fentanyl 25 mcg IV q5 minutes PRN for breakthrough pain (PACU only); hydromorphone 0.5-1.0 mg IV q3-4 hour for breakthrough pain after discharge from PACU	Ketorolac 30 mg IV q6 hours x4 doses (upon PACU arrival); celecoxib 200 mg PO BID (beginning evening of POD1); oxycodone 5/10 mg PO q3 hours PRN for moderate/severe pain; hydromorphone 0.2/0.4 mg IV q5 minutes PRN for moderate/severe pain (PACU only); fentanyl 25 mcg IV q5 minutes PRN for breakthrough pain (PACU only); hydromorphone 0.5-1.0 mg IV q3-4 hour for breakthrough pain after discharge from PACU

TKA	Diclofenac cohort	Ketorolac cohort
Preoperative	Celecoxib 400 mg PO (dose-adjusted for age and/or renal dysfunction) ±spinal anesthesia (with morphine and bupivacaine)	Celecoxib 400 mg PO (dose-adjusted for age and/or renal dysfunction) ±spinal anesthesia (with morphine and bupivacaine)
Intraoperative	Periarticular multimodal drug injection containing bupivacaine, epinephrine, dexamethasone, ketorolac, and morphine	Periarticular multimodal drug injection containing bupivacaine, epinephrine, dexamethasone, ketorolac, and morphine
Postoperative	Adductor canal block (ACB) containing bupivacaine and epinephrine ±dexamethasone; diclofenac sodium 37.5 mg IV q6 hours x4 doses (upon PACU arrival); celecoxib 200 mg PO BID (beginning evening of POD1); oxycodone 5/10 mg PO q3 hours PRN for moderate/severe pain; hydromorphone 0.2/0.4 mg IV q5 minutes PRN for moderate/severe pain (PACU only); fentanyl 25 mcg IV q5 minutes PRN (PACU only); hydromorphone 0.5-1.0 mg IV q3-4 hour for breakthrough pain after discharge from PACU	Adductor canal block (ACB) containing bupivacaine and epinephrine ±dexamethasone; ketorolac 30 mg IV q6 hours x4 doses (upon PACU arrival); celecoxib 200 mg PO BID (beginning evening of POD1); oxycodone 5/10 mg PO q3 hours PRN for moderate/severe pain; hydromorphone 0.2/0.4 mg IV q5 minutes PRN for moderate/severe pain (PACU only); Fentanyl 25 mcg IV q5 minutes PRN (PACU only); hydromorphone 0.5-1.0 mg IV q3-4 hour for breakthrough pain after discharge from PACU

In addition, the diclofenac group received 37.5 mg of IV diclofenac sodium every six hours for four doses, beginning on arrival to the postoperative anesthesia care unit (PACU). The ketorolac group similarly received 30 mg of IV ketorolac every six hours for four doses upon arrival to the PACU. Oxycodone immediate-release (5 or 10 mg PO) was made available postoperatively to all patients in both groups to ease breakthrough pain if necessary.

Study variables

Demographic information including gender, race, age, and body mass index (BMI) was collected for all patients. Opioid consumption [converted to morphine milligram equivalents [MME]), length of stay (LOS), visual analog scale (VAS) pain scores at eight-hour intervals, discharge destination, and post-admission satisfaction scores were collected to evaluate patient outcomes. Patient satisfaction with postoperative pain control was assessed via three questions selected from the Hospital Consumer Assessment of Healthcare Providers and Systems (HCAHPS) survey (Table 2) [25].

**Table 2:** Hospital Consumer Assessment of Healthcare Providers and Systems patient satisfaction questions

Question	Answer
During this hospital stay, how well was your pain controlled?	1: Never (least satisfied); 2: Sometimes; 3: Usually; 4: Always (most satisfied)
During this hospital stay, how often was your pain well-controlled?	
During this hospital stay, how often did the hospital staff do everything they could to help you with your pain?	

Pain intensity, expressed as area under the curve (AUC), was calculated utilizing VAS pain scores at eight-hour intervals through the 48-hour postoperative period.

The THA patients in the IV diclofenac and IV ketorolac cohorts differed in race (54.2 vs. 82.1% white; p: 0.029) and gender (33.3 vs. 60.7% female; p: 0.049); however, there were no differences in other demographic characteristics, including age (57.25 vs. 58.86 years; p: 0.641) and BMI (30.66 vs. 28.81 kg/m2; p: 0.297) (Table 3).

**Table 3:** Demographic characteristics in the IV diclofenac and IV ketorolac groups THA: total hip arthroplasty; TKA: total knee arthroplasty; IV: intravenous; min: minute; n: number; SD: standard deviation

	THA			TKA		
	Diclofenac	Ketorolac	P-value	Diclofenac	Ketorolac	P-value
Total number	25	28		51	32	
Gender, n (%)						
Female	8 (33.3%)	17 (60.7%)		34 (66.7%)	20 (62.5%)	
Male	16 (66.7%)	11 (39.3%)	0.049	17 (33.3%)	12 (37.5%)	0.698
Race, n (%)						
White	13 (54.2%)	23 (82.1%)		18 (35.3%)	17 (53.1%)	
Black	11 (45.8%)	5 (17.9%)		32 (62.7%)	15 (46.9%)	
Indian	0 (0%)	0 (0%)	0.029	1 (2.0%)	0 (0%)	0.225
Mean age at surgery, years (±SD)	57.25 (±11.96)	58.86 (±12.58)	0.641	57.25 (±11.96)	58.86 (±12.58)	0.641
Mean BMI, kg/m^2^, (±SD)	30.66 (±7.34)	28.81 (±5.27)	0.297	34.5 (±6.11)	33.3 (±6.86)	0.430
Mean operative time, min, (±SD)	121.52 (±29.18)	118.96 (±36.42)	0.786	130 (±30.6)	128 (±36.7)	0.849
Anesthesia type, n (%)						
Spinal	16 (66.7%)	24 (85.7%)		2 (3.9%)	1 (3.1%)	
Spinal + general	3 (12.5%)	1 (3.6%)		41 (80.4%)	31 (96.9%)	
General	5 (20.8%)	3 (10.7%)	0.245	8 (15.7%)	0 (0%)	0.059

Baseline demographic characteristics including age, BMI, gender, race, and anesthesia type were similar between the two TKA groups (p: >0.05 for all).

Statistical analysis

Chi-square analysis/Fisher’s exact test and Student’s t-test were utilized to analyze categorical and continuous variables, respectively. A p-value of less than 0.05 was designated as statistically significant. All statistical analysis was performed using IBM SPSS Statistics version 24 (IBM Corporation, Armonk, NY).

## Results

For THA recipients, there was no significant difference between the IV diclofenac and ketorolac groups in mean total morphine milliequivalents (MME) opioid consumption at 12 (24.48 vs. 32.02 mg; p: 0.227) or 24 hours (17.71 vs. 18.25 mg; p: 0.915) postoperatively (Table 4).

**Table 4:** Differences in pain control outcomes between IV diclofenac and IV ketorolac THA patients THA: total hip arthroplasty; MME: morphine milliequivalents; S.D.: standard deviation; AUC: area under the curve; IV: intravenous

THA	Diclofenac	Ketorolac	P-value
Mean opioid consumption, MME (±SD)			
MME 0-12 hours (±SD)	24.48 (±21.06)	32.02 (±23.01)	0.227
MME 12-24 hours (±SD)	17.71 (±16.57)	18.25 (±19.48)	0.915
Mean pain intensity (AUC)			
AUC 24 hours (±SD)	80.00 (±34.04)	95.00 (±47.24)	0.202
AUC 48 hours (±SD)	68.50 (±47.57)	63.57 (±49.07)	0.716

There were also no differences found in postoperative pain intensity POD1 (80.00 vs. 95.00; p: 0.202) or POD2 (68.50 vs. 63.57; p: 0.716), LOS (2.00 vs. 2.18 days; p: 0.135), or discharge destination (87.5 vs. 89.3% home; p: 0.217) (Table 5). However, patients who received diclofenac were more satisfied with their pain control than those given ketorolac (Table 6).

**Table 5:** Postoperative outcomes in IV diclofenac and IV ketorolac THA patients THA: total hip arthroplasty; SD: standard deviation; IV: intravenous

THA	Diclofenac	Ketorolac	P-value
Mean length of stay, days (±SD)	2.00 (±0.417)	2.18 (±1.79)	0.135
Discharge destination, n (%)			0.217
Home	21 (87.5%)	25 (89.3%)	
Inpatient rehab	2 (8.3%)	0 (0%)	
Acute rehab	1 (4.2%)	3 (10.7%)	

**Table 6:** Patient satisfaction scores in IV diclofenac and IV ketorolac THA patients THA: total hip arthroplasty; IV: intravenous *Posthoc analysis (adjusted residuals)

THA	Diclofenac	Ketorolac	P-value
Patient satisfaction question 1: “During this hospital stay, how well was your pain controlled?”			
Always, n (%)	18 (75.0%)	12 (48.0%)	0.021
Usually, n (%)	5 (20.8%)	4 (16.0%)	
Sometimes*, n (%)	1 (4.2%)	9 (36.0%)	
Patient satisfaction question 2: “During this hospital stay, how often was your pain well-controlled?”			
Always*, n (%)	19 (79.2%)	12 (48.0%)	0.019
Usually, n (%)	4 (16.7%)	4 (16.0%)	
Sometimes*, n (%)	1 (4.2%)	9 (36.0%)	
Patient Satisfaction Question 3: “During this hospital stay, how often did the hospital staff do everything they could to help you with your pain?”			
Always*, n (%)	23 (95.8%)	12 (48.0%)	0.001
Usually, n (%)	1 (4.2%)	4 (16.0%)	
Sometimes*, n (%)	0 (0%)	9 (36.0%)	

Specifically, IV diclofenac recipients answered “Always” significantly more often than IV ketorolac patients in response to the questions “During this hospital stay, how well was your pain controlled?” (75.0 vs. 48.0%; p: 0.021); “During this hospital stay, how often was your pain well-controlled?” (79.2 vs. 48.0%; p: 0.019); and “During this hospital stay, how often did the hospital staff do everything they could to help you with your pain?” (95.8 vs. 48.0%; p: 0.001).

For TKA recipients, patients in the IV diclofenac cohort had lower opioid consumption than patients in the IV ketorolac cohort during the first twelve hours postoperatively (21.6 vs. 32.8 MME; p: 0.037) (Table 7).

**Table 7:** Differences in pain control outcomes between IV diclofenac and IV ketorolac TKA patients TKA: total knee arthroplasty; MME: morphine milliequivalents; SD: standard deviation; AUC: area under the curve; IV: intravenous

TKA	Diclofenac	Ketorolac	P-value
Mean opioid consumption, MME (±SD)			
MME 0-12 hours (±SD)	21.6 (±19.8)	32.8 (±28.0)	0.037
MME 12-24 hours (±SD)	19.0 (±19.7)	21.5 (±21.8)	0.596
Mean pain intensity (AUC)			
AUC 24 hours (±SD)	81.6 (±43.8)	93.6 (±49.1)	0.251
AUC 48 hours (±SD)	85.5 (±48.0)	84.3 (±51.3)	0.911

There were no differences between the diclofenac and ketorolac groups in opioid consumption at 24 hours (19.0 vs. 21.5 MME; p: 0.596), pain intensity on POD1 (81.6 vs. 93.6; p: 0.251) or POD2 (85.5 vs. 84.3; p: 0.911), or mean LOS (2.6 vs. 2.1 days; p: 0.063). The diclofenac group was less likely than the ketorolac group to be discharged home (58.8 vs. 84.4% home; p: 0.025) (Table 8).

**Table 8:** Postoperative outcomes in IV diclofenac and IV ketorolac TKA patients TKA: total knee arthroplasty; SD: standard deviation; KSS: Knee Society Score; IV: intravenous *Posthoc analysis (adjusted residuals)

TKA	Diclofenac	Ketorolac	P-value
Mean length of stay, days (±SD)	2.63 (±1.82)	2.09 (±0.69)	0.063
Discharge destination, n (%)			0.025
Home*	30 (58.8%)	27 (84.4%)	
Inpatient rehab*	9 (17.6%)	0 (0.0%)	
Acute rehab	8 (15.7%)	4 (12.5%)	
Subacute rehab	4 (7.8%)	1 (3.1%)	
Difference in KSS score at 1 month, mean (±SD)	20.0 (±6.62)	22.7 (±5.61)	0.068

There was no difference in mean postoperative Knee Society Score (KSS) between the groups (20.0 vs. 22.7; p: 0.068). Patients receiving diclofenac were significantly more satisfied with their pain control experience than those who received ketorolac (Table 9).

**Table 9:** Patient satisfaction scores in IV diclofenac and IV ketorolac TKA patients TKA: total knee arthroplasty; IV: intravenous *Posthoc analysis (adjusted residuals)

TKA	Diclofenac	Ketorolac	P-value
Patient satisfaction question 1: “During this hospital stay, how well was your pain controlled?”			
Always*, n (%)	37 (74.0)	12 (46.2%)	<0.001
Usually, n (%)	13 (26.0%)	5 (19.2%)	
Sometimes*, n (%)	0 (0%)	9 (34.6%)	
Patient satisfaction question 2: “During this hospital stay, how often was your pain well-controlled?”			
Always*, n (%)	35 (70.0%)	12 (46.2%)	<0.001
Usually, n (%)	15 (30%)	5 (19.2%)	
Sometimes*, n (%)	0 (0%)*	9 (34.6%)*	
Patient satisfaction question 3: “During this hospital stay, how often did the hospital staff do everything they could to help you with your pain?”			
Always*, n (%)	48 (96.0%)	12 (46.2%)	<0.001
Usually*, n (%)	2 (4.0)	6 (23.1%)	
Sometimes*, n (%)	0 (8%)	8 (30.8%)	

Specifically, IV diclofenac recipients answered “Always” more often than IV ketorolac patients in response to the questions “During this hospital stay, how well was your pain controlled?” (74.0 vs. 46.2%; p: <0.001); “During this hospital stay, how often was your pain well-controlled?” (70.0 vs. 46.2%; p: <0.001); and “During this hospital stay, how often did the hospital staff do everything they could to help you with your pain?” (96.0 vs. 46.2%; p: <0.001).

## Discussion

Optimizing pain control regimens following TJA is an important aspect to help these procedures transition to the outpatient setting, as improved pain management can improve clinical outcomes and curtail costs [26]. Due to their efficacy in pain relief and in reducing opioid consumption, NSAIDs such as diclofenac and ketorolac have emerged as a promising adjunctive pain control modalities [16,20]. The present study demonstrated that while primary TKA patients receiving IV diclofenac had significantly lower opioid consumption during the first 12 hours postoperatively, there was no difference in opioid consumption at 24 hours when compared to those who received IV ketorolac. In addition, there were no significant differences in opioid consumption or discharge destination in THA patients and no differences in postoperative pain intensity in both TKA and THA patients. However, TKA and THA patients on the IV diclofenac regimen were significantly more satisfied with their postoperative pain experience than those on the IV ketorolac regimen. The IV formulation of both these nonopioid medications appears effective in managing postoperative TJA pain and may be useful in allowing patients to have a faster discharge.

There are some limitations to this study. As we only included procedures performed by a single surgeon at a single institution, the sample sizes are small. However, this was meant to be a preliminary study, reporting the early outcomes of IV NSAIDs in TJA. Additionally, data input and collection may have been susceptible to human error. To minimize this risk, data collection was limited to one investigator and the same nursing floor. Finally, satisfaction was significantly higher for the diclofenac group, despite similar outcomes. This could be due to the small sample sizes, as patients were blinded, and pain intensity was similar in all groups. Nevertheless, the present study is a great starting point for the investigation of TJA outcomes in patients receiving adjunctive IV diclofenac and IV ketorolac.

Although TKA patients receiving IV diclofenac had lower opioid consumption at 12 hours, our findings revealed no difference in 24-hour opioid consumption for TKA and THA patients in the IV diclofenac and IV ketorolac groups. Other studies have reported on the comparative decrease in opioid consumption with both IV diclofenac and IV ketorolac. A double-blind, randomized control trial by Alexander et al. compared morphine consumption, as well as pain scores and opioid side effects, in 102 TKA and THA patients who were given IV diclofenac, IV ketorolac, or placebo preoperatively [27]. They revealed that diclofenac and ketorolac patients both consumed significantly less morphine than the placebo group (p: 0.032), and experienced significantly less opioid side effects (p: <0.05). In addition, George et al. compared adjunctive IV diclofenac to standard pain management in a retrospective study of 113 THA patients, finding a significant reduction in opioid consumption on POD1 and 2 for IV diclofenac patients (p: 0.001) [24]. Furthermore, another study of 52 TKAs by George et al. assessed the addition of IV diclofenac to a multimodal pain control regimen and demonstrated a significant reduction in opioid consumption among patients who received IV diclofenac (p: 0.041) [23]. These results show the efficacy of IV NSAIDs in reducing the need for postoperative opioids, an important goal in the current fight to minimize opioid misuse and potential addiction.

The present study demonstrated no differences in postoperative pain intensity or LOS between the IV diclofenac and IV ketorolac THA and TKA cohorts. Although prior studies have found decreased postoperative pain with the use of ketorolac and diclofenac in TJA, no study has demonstrated a significant difference between the two drugs. Etches et al. performed a multicenter, double-blinded, randomized controlled trial (RCT) comparing postoperative pain control between TKA and THA patients who received general anesthesia with placebo (n = 88) or with continuous infusion ketorolac (n = 86) [28]. Compared to placebo, the ketorolac cohort had significantly decreased pain intensity at two, four, and six hours postoperatively (p: <0.05 for all). A double-blind study by Minotti et al. demonstrated similar results, finding that in 180 patients with cancer pain, 10 mg of intramuscular ketorolac was equivalent in analgesic efficacy to 75 mg of intramuscular diclofenac [29]. Moreover, a prospective, double-blinded comparative study by Kostamovaara et al. randomized primary THA patients (n = 85) to receive ketorolac (n = 28), diclofenac (n = 28), or ketoprofen (n = 29) [20]. The authors reported no difference between groups in median VAS scores up through sixteen hours postoperatively (p: >0.05 for all time points). These studies all corroborate our results and reinforce the ability of NSAIDs, particularly diclofenac and ketorolac, to provide adequate analgesia and have comparable LOS. These medications also appear well suited for utilization in the outpatient setting, as they are most efficacious in the first 24 hours after surgery.

Although patients in both THA and TKA diclofenac cohorts had increased satisfaction with IV diclofenac over IV ketorolac, outcomes were similar between the cohorts. Similar postoperative pain intensities and opioid consumption demonstrate the effectiveness of both drugs in managing postoperative pain. However, there are no studies comparing the cost of IV diclofenac to IV ketorolac for orthopedic surgery. In our institution, there is a vast pricing difference between IV diclofenac and IV ketorolac. The cost of four doses of IV diclofenac is USD 48.00, while the cost of four doses of IV ketorolac is USD 3.12. When extrapolated for 1,000 patients, the cost savings between these two drugs becomes apparent (USD 44,880 saved per 1,000 patients). This supports the utilization of IV ketorolac in postoperative pain management to yield the greatest cost savings. Nonetheless, the utilization of IV NSAIDs for pain management after TJA appears beneficial in reducing opioid consumption and may be well suited to supporting outpatient surgery in the future.

## Conclusions

Optimizing postoperative pain management is essential to improving clinical outcomes and reducing costs, especially as TJA moves out of the hospital. The addition of NSAIDs to the postoperative pain regimen is a promising way of decreasing opioid consumption and reducing postoperative pain. This study demonstrated that both TKA and THA patients treated with IV diclofenac had no difference in postoperative pain intensity while THA patients had no difference in opioid consumption relative to those treated with IV ketorolac. Further comparison of IV NSAIDs with other IV pain medications may provide broader insight into the ideal management of postoperative pain for this widening patient population.
